# Influence of bladder fullness on the detection of urinary tract obstruction by dynamic renal scintigraphy

**DOI:** 10.1590/0100-3984.2016-0061

**Published:** 2017

**Authors:** Nathalia Novaes Cosenza, Fábio Lau, Mariana Cunha Lopes Lima, Barbara Juarez Amorim, Camila Mosci, Marcelo Lopes Lima, Celso Darío Ramos

**Affiliations:** 1 MD, Resident in Nuclear Medicine, Faculdade de Ciências Médicas da Universidade Estadual de Campinas (FCM-Unicamp), Campinas, SP, Brazil.; 2 Medical Student, Faculdade de Ciências Médicas da Universidade Estadual de Campinas (FCM-Unicamp), Campinas, SP, Brazil.; 3 PhD, Attending Nuclear Medicine Physician, Faculdade de Ciências Médicas da Universidade Estadual de Campinas (FCM-Unicamp), Campinas, SP, Brazil.; 4 MSc, Attending Nuclear Medicine Physician, Faculdade de Ciências Médicas da Universidade Estadual de Campinas (FCM-Unicamp), Campinas, SP, Brazil.; 5 PhD, Urologist, Faculdade de Ciências Médicas da Universidade Estadual de Campinas (FCM-Unicamp), Campinas, SP, Brazil; 6 PhD, Professor of Nuclear Medicine, Faculdade de Ciências Médicas da Universidade Estadual de Campinas (FCM-Unicamp), Campinas, SP, Brazil.

**Keywords:** Dynamic renal scintigraphy, Urinary tract obstruction, ^99m^Tc-DTPA, Vesical repletion.

## Abstract

**Objective:**

To investigate the influence of bladder fullness on the diagnosis of urinary tract obstruction during dynamic renal scintigraphy with a diuretic stimulator.

**Materials and methods:**

We studied 82 kidneys in 82 patients submitted to dynamic renal scintigraphy with a diuretic stimulator. We compared the proportional elimination of the radiopharmaceutical ^99m^Tc-DTPA from the kidneys before and after bladder emptying in post-diuretic images, classifying each image as representing an obstructed, indeterminate, or unobstructed kidney.

**Results:**

The overall elimination of ^99m^Tc-DTPA from the kidneys was 10.4% greater after bladder emptying than before. When the analysis was performed with a full bladder, we classified 40 kidneys as obstructed, 16 as indeterminate, and 26 as unobstructed. When the 40 kidneys classified as obstructed were analyzed after voiding, 11 were reclassified as indeterminate and 3 were reclassified as unobstructed. Of the 16 kidneys classified as indeterminate on the full-bladder images, 13 were reclassified as unobstructed after voiding.

**Conclusion:**

In dynamic renal scintigraphy with a diuretic stimulator, it is important to obtain images after voiding, in order to perform a reliable analysis of the proportional excretion of ^99m^Tc-DTPA from the kidneys, avoiding possible false-positive results for urinary tract obstruction.

## Abstract (pt)

**Objetivo:**

Verificar a influência da repleção vesical no diagnóstico da obstrução do trato urinário durante a cintilografia renal dinâmica com estímulo de diurético.

**Materiais e métodos:**

Foram estudados, retrospectivamente, 82 rins de 82 pacientes submetidos a cintilografia renal dinâmica. Compararam-se as porcentagens de excreção do radiofármaco DTPA-^99m^Tc pelos rins antes e após o esvaziamento vesical nas imagens pós-diurético, classificando-os como obstruídos, indeterminados ou não obstruídos.

**Resultados:**

A avaliação da excreção do radiofármaco pelos rins mostrou que houve aumento de 10,4% na taxa de excreção global quando a bexiga foi esvaziada. Dos 82 rins estudados, 40 foram considerados obstruídos, 16 indeterminados e 26 como não obstruídos, na análise com a bexiga repleta. Na análise das imagens após micção, dos 40 classificados como obstruídos, 11 passaram a ser classificados como indeterminados e 3 como não obstruídos. Além disso, dos 16 rins apontados como indeterminados nas imagens com a bexiga repleta, 13 passaram a ser considerados não obstruídos com a bexiga vazia.

**Conclusão:**

É fundamental uma imagem após a micção na cintilografia renal dinâmica para uma análise fidedigna da porcentagem de excreção do radiofármaco pelo rim, evitando-se possíveis falso-positivos para obstrução do trato urinário.

## INTRODUCTION

Urinary tract obstruction (UTO) is a relatively common clinical condition in various age groups and can be defined as partial or total restriction of urinary flow that can result in kidney injury and renal failure^(1)^. Upper UTO results in back pressure in the tubules and vessels within the pelvis, together with increased peristaltic activity, resulting in dilatation of the system and uncoordinated peristalsis. Acute obstruction can be accompanied by symptoms, whereas chronic obstruction is typically silent^(2,3)^.

UTO is a leading cause of renal dysfunction. To determine the most appropriate treatment, it is extremely important to differentiate between mechanical obstruction, as in the case of ureteropelvic junction anomalies, and non-obstructive dilatation, as occurs in non-obstructive hydronephrosis^(4)^. Non-obstructive hydronephrosis can be caused by reflux, primary megaureter, or previously resolved obstruction. Neonatal hydronephrosis is commonly identified through imaging studies during pregnancy and can have an obstructive origin, often caused by obstruction at the ureteropelvic junction or ureterovesical junction, or a non-obstructive origin. The distinction between those two origins plays an important role in the decision-making process regarding the clinical management of the condition^(5)^.

OTU can be evaluated by the Whitaker test and by dynamic renal scintigraphy (DRS). However, although they have the same objective, each of those methods has its peculiarities^(6,7)^.

The urodynamic Whitaker test evaluates pelvic urinary tract pressure during increasing infusion of fluid, necessitating percutaneous nephrostomy. Because it is a relatively invasive and non-physiological examination, the Whitaker test is reserved for special cases, such as those of patients with a markedly dilated urinary tract and diminished renal function, as well as those in which the results of scintigraphy with a diuretic stimulator are inconclusive or the patient already has a nephrostomy tube in place^(6,7)^.

DRS is used not only to study obstructions in the urinary tract but also to evaluate megaureter, horseshoe kidney, polycystic kidney, ectopic ureterocele, postoperative states, pyeloplasty, ureteral reimplantation, and other conditions^(2)^. The examination takes on even more importance in the evaluation of pediatric patients, among whom it is typically more difficult to make the correct clinical diagnosis^(8)^. The scintigraphic evaluation is made through visual analysis and on the basis of several parameters that directly or indirectly quantify elimination of the radiopharmaceutical by the urinary route^(9)^. DRS involves venous administration of a radiopharmaceutical, such as technetium-99m-labeled mercaptoacetyltriglycine (^99m^Tc-MAG3)^(10,11)^, technetium-99m-labeled ethylenedicysteine (^99m^Tc-EC)^(12,13)^, and technetium-99m-labeled diethylenetriaminepentaacetic acid (^99m^Tc-DTPA)^(3,14)^. The last is the most widely used in many countries, due to its ease of preparation, availability, and low cost^(15)^. The tracer is taken up and then excreted by the kidneys. The DRS images are obtained with a scintillation camera. If there is obstruction, the tracer is retained in the upper urinary tract, showing that the urine flow is low, even with the use of a diuretic stimulator^(14)^. If there is no obstruction, the tracer will flow into the bladder together with the urine^(14)^.

To make the study more accurate and reproducible, the amount of radioactive material eliminated after administration of the diuretic is typically quantified by measuring the half-time (T½) to clearance of the material^(8,16)^ or by measuring the proportional elimination following administration of the diuretic^(15)^. The simple fact of not emptying the bladder during DRS can alter the outcome of the examination, because a full bladder can increase the pressure in the upper urinary tract, preventing urine from flowing into the ureter, which can lead to a false-positive result for obstruction^(14)^.

To keep the bladder empty, various authors have recommended catheterization of the bladder throughout the procedure^(8,16)^. Other authors consider routine bladder catheterization inappropriate, because it makes the study unnecessarily invasive, promoting the occurrence of urinary infection. The alternative is to interrupt the examination for a few minutes before and after administration of the diuretic, so that the patient can, at those two time points, empty the bladder naturally and static post-micturition images can be acquired, which allows the proportional elimination to be calculated^(15)^. The objective of the present study was to determine the influence of bladder fullness on the diagnosis of upper UTO during DRS involving the use of furosemide as a diuretic stimulator. We also tested the hypothesis that a full bladder complicates the drainage of the renal pelvis and ureter, as well as promoting false-positive results.

## MATERIALS AND METHODS

This was a retrospective study involving 82 consecutive patients (39 men and 43 women), ranging in age from 1 month to 83 years (mean, 25.77 ± 22.99 years; median, 15 years). Patients were selected from among those referred to the department of nuclear medicine for DRS with ^99m^Tc-DTPA involving the administration of a diuretic (furosemide), due to suspicion of UTO, between June 2012 and February 2015. Diuretic administration of the diuretic was indicated when retention of the radiopharmaceutical in the renal pelvis or renal pelvis/ureter was seen on the post-micturition image obtained after the dynamic study. New pre- and post-micturition images (dynamic and static) were then acquired. Among the 82 patients in the study sample, 94 kidneys were initially evaluated, due to the suspicion of bilateral UTO raised by the scintigraphy findings in 12 of the patients. To avoid statistical bias in those 12 cases, the proportional elimination of the radiopharmaceutical was calculated for each kidney, and only the kidney with less elimination was chosen, resulting in the analysis of only one dilated kidney per patient.

Examinations performed with a radiopharmaceutical other than ^99m^Tc-DTPA, such as ^99m^Tc-EC, were excluded, as were those of patients for whom the data were incomplete, patients who did not take the furosemide test, patients who for any reason did not follow the usual protocol, and patients in whom bladder catheterization was employed, because that would preclude evaluation of the influence of bladder fullness.

### Preparation of the radiopharmaceutical

The lyophilized reagent kit used in order to prepare the ^99m^Tc-DTPA (Instituto de Pesquisas Energéticas e Nucleares, São Paulo, Brazil) was reconstituted according to the manufacturer's instructions. The ^99m^Tc sodium pertechnetate used in the DTPA labeling was obtained from molybdenum-99/technetium-99m generators (Instituto de Pesquisas Energéticas e Nucleares).

The reaction flask contained a lyophilized mixture of 10 mg of DTPA, 1.0 mg of stannous chloride dehydrate, and 2.0 mg of para-aminobenzoic acid. The labeling with technetium-99m was performed by adding to the reaction flask a quantity of ^99m^Tc sodium pertechnetate sufficient to produce a maximum activity of 3,700 MBq (100 mCi), diluted with saline solution to a volume of 3 mL. The flask was gently agitated for 10 s, inverted several times for 10 s each, and left at room temperature for 15 min to complete the reaction. The standard dose used for adults was 20 mCi of ^99m^Tc-DTPA, which was adjusted for the children in the sample, by weight and age, according to the dose table (paediatric dosage card, version 01.02.2014) devised by the European Association of Nuclear Medicine^(17)^. The labeling control was performed by paper chromatography, acceptability being defined as a labeling efficiency ≥ 90%.

### Preparation for the examination

Patients were instructed to drink 500 mL of water 1 h before the start of the test, the exceptions being infants and children under 2 years of age, for whom *ad libitum* liquid intake was advised. All of the patients were instructed to empty their bladder immediately prior to the start of the examination.

### Acquisition protocol for renal scintigraphy with ^99m^Tc-DTPA

The examinations were performed in scintillation cameras (Millennium system; GE Healthcare, Haifa, Israel, and Symbia; Siemens, Hoffman Estates, IL, USA) equipped with low-energy general use collimators.

Dynamic images were acquired over a 25-min period with the patient in the supine position, in the posterior projection of the abdomen with a 64 × 64 matrix and variable zoom depending on patient size, so that the kidneys and bladder were included in the field of view. Dynamic image acquisition was initiated immediately after the administration of the radiopharmaceutical as an intravenous bolus injection and consisted of two phases, one in which one image was obtained every 2 s for 80 s (blood flow phase) and another in which one image was obtained every 15 s for 25 min (functional phase). Static images were then acquired in the same projection and at the same zoom for 60 s, before and after bladder emptying. After urination, the diuretic furosemide (40 mg for adults and 1 mg/kg for children, at a maximum dose of 40 mg) was administered intravenously, and new dynamic images (in the same projection and with the same acquisition parameters as the first dynamic images) were acquired over another 20-min period. New static images (in the same projection and with the same acquisition parameters as the first static images) were also obtained, before and after new bladder emptying, in order to calculate the proportional elimination of the radiopharmaceutical after administration of the diuretic. At the end of the acquisition of the first dynamic images, a static image was also acquired in the anterior projection of the head and neck region for 60 s, with variable zoom depending on the size of the patient, in order to rule out the potential *in vivo* unlabeling of the radiopharmaceutical, identified by uptake of free ^99m^Tc sodium pertechnetate by the thyroid and salivary glands. In the range of pH 3.5-4.5, the labeling efficiency was > 90%.

### Image processing

The images were processed on the consoles of the equipment used in their acquisition. The analyses were performed by delineating regions of interest (ROIs) around each kidney and the aorta in the dynamic images obtained during the flow phase, calculating time-activity curves, and representing the blood radioactivity counts for each kidney versus the time in seconds. The dynamic images obtained every 2 min during the functional phase were grouped, and ROIs were drawn around each kidney and their collecting systems in the image for 2- to 3-min intervals. The background radiation was subtracted using automatically defined ROIs around the outer perimeter of the ROI for each kidney. The data obtained allowed the relative glomerular function to be quantified and the renogram (time-activity curves for each kidney, representing the radioactivity counts for each kidney versus the time in seconds) to be obtained.

The proportional elimination of ^99m^Tc-DTPA by the kidneys was calculated by tracing ROIs around the renal pelvis, collecting systems, and ureters (the last only when there was ureteral retention of the radiopharmaceutical), in the following images: static pre- and post-micturition images before administration of the diuretic (the image with the higher radioactivity count, generally the premicturition image, being chosen for quantification); static images obtained after administration of the diuretic with a full bladder; and static images obtained after administration of the diuretic and new bladder emptying. The proportional elimination of the radiopharmaceutical was calculated with the following equation:

$$E=\left(A_{1}-A_{2}\right)\times 100/A_{I}$$

where *E* is the proportional post-diuretic elimination, *A_1_* is pre-diuretic radioactivity, and *A_2_* is post-diuretic radioactivity. This calculation was made twice: once using the *A_2_* obtained from the post-diuretic image with a full bladder; and once using the *A_2_* obtained from the post-diuretic image with an empty bladder.

Post-diuretic time-activity curves (radioactivity counts of the excretory pathways of each kidney versus the time in seconds) were also obtained by plotting ROIs around the renal pelvis, collecting systems, and ureters (the last only when there was ureteral retention of the radiopharmaceutical) in the dynamic images obtained 20 min after administration of furosemide.

### Qualitative and semiquantitative analyses

Qualitative visual analysis was performed by evaluating renal blood flow, together with renal accumulation, concentration, and excretion of the radiopharmaceutical. The qualitative analysis of the blood flow phase used the abdominal aorta as a reference. Renal blood flow was considered normal when, within 6 s of the radioactivity peak in the aorta, the radioactivity peak in the kidney was greater than that recorded for the aorta. The analysis of the functional phase was also performed qualitatively, by evaluating the images and the renogram curves. The analysis included the accumulation phase, in which we evaluated extraction of the radiopharmaceutical from the bloodstream in the first 3 min; the concentration phase, in which we evaluated the urinary concentrating ability (water reabsorption capacity); and the excretion phase, in which we evaluated the transport of the radiopharmaceutical to the bladder by the renal pelvis system and ureters. The classification of glomerular function was classified, also by visual analysis, as normal, discrete, moderate, or markedly depressed according to the degree to which accumulation and concentration of the radiopharmaceutical was reduced. All analyses were performed by the same operator and evaluated by two nuclear physicians.

The evaluation of the proportion of ^99m^Tc-DTPA eliminated was adapted from the T½ method, as previously described^(15)^. The test result was considered indicative of an obstructive kidney if the proportional elimination was < 50% at 20 min, corresponding to a T½ > 20 min; indicative of an unobstructed kidney if the proportional elimination was ≥ 60% at 20 min, corresponding to a T½ < 15 min; or undetermined if the proportional elimination was 50-60% at 20 min^(12)^. Those values were used for adult and pediatric patients, as previously described^(12)^.

### Statistical analysis

We evaluated the proportional post-diuretic elimination of the radiopharmaceutical from kidneys classified as suspect, comparing the pre- and post-micturition images. We counted the number of kidneys that evolved from obstructed to undetermined or unobstructed, that evolved from undetermined to unobstructed, and that did not change after bladder emptying. We also analyzed the influence that age-as a continuous variable and as a dichotomous variable (> vs. ≤ 5 years of age)-and gender have on UTO. We compared the two age groups (> 5 years of age and ≤ 5 years of age), in terms of the proportional retention, through repeated-measures analysis of variance, including the influence of gender. The comparison between the groups by category of proportional retention was made by the test of symmetry.

The level of significance adopted was 5%. Statistical analysis of the data was performed with the Statistical Analysis System for Windows, version 9.4 (SAS Institute, Cary, NC, USA).

## RESULTS

The analysis of overall excretion of the radiopharmaceutical by the 82 kidneys evaluated showed that the mean proportional elimination of ^99m^Tc-DTPA per kidney in the presence of the a full bladder was 44.30% ± 28.03%, ranging from −57.10% to 83.60%. The cases in which the proportional elimination was negative were attributable to additional uptake of the radiopharmaceutical during the acquisition of the post-diuretic images. The same analysis performed with the post-micturition images showed that the mean proportional elimination of the radiopharmaceutical was 54.70% ± 25.56%, ranging from −52.94% to 88.36%. Therefore, the overall excretion rate was 10.4% higher when the bladder was empty, a statistically significant difference (*p* < 0.001).

**Figure 1:**
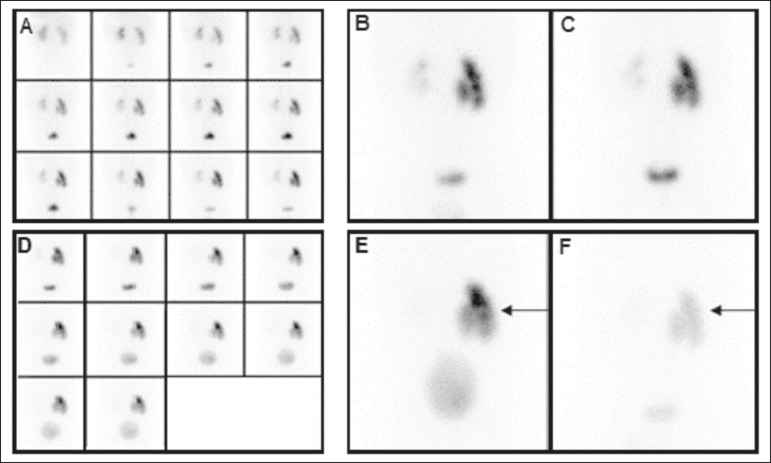
DRS with ^99m^Tc-DTPA and diuretic administration in a 4-month-old female patient with suspected obstruction of the right kidney. Dynamic images obtained every 2 min and grouped (**A**), static pre-micturition images (**B**), and static post-micturition images (**C**), all demonstrating retention of the radiopharmaceutical in the right renal pelvis. New dynamic images and new static pre-micturition images, both obtained after intravenous injection of a diuretic (**D** and **E**, respectively), also show retention of the radiopharmaceutical in the right renal pelvis, with proportional elimination of −11%, the negative proportion indicating an increase in the amount of material retained (arrow). Post-diuretic, post-micturition images (**F**) showed satisfactory excretion of the material retained in the right kidney (arrow), with a proportional elimination of 63%. Therefore, the result was changed from "obstructed" to "unobstructed".

**Figure 2:**
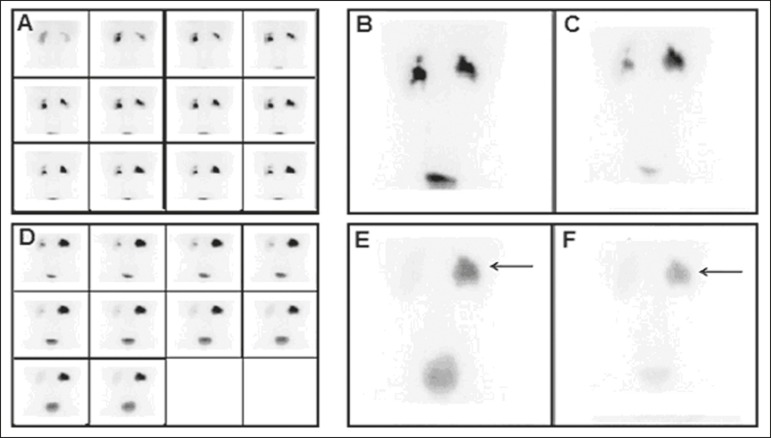
DRS with ^99m^Tc-DTPA and diuretic administration in a 13-year-old male patient with suspected obstruction of the right kidney. Dynamic images obtained every 2 min and grouped (**A**), static pre-micturition images (**B**), and static post-micturition images (**C**), all demonstrating marked retention of the radiopharmaceutical in the right renal pelvis. New dynamic images and new static pre-micturition images, both obtained after intravenous injection of a diuretic (**D** and **E**, respectively), also show retention of the radiopharmaceutical in the right renal pelvis, with proportional elimination of 24% (arrow). Post-diuretic, post-micturition images (**F**) showed additional excretion of the material retained in the right kidney (arrow), with a proportional elimination of 51%. In this case, the result was changed from "obstructed" to "indeterminate".

In the post-diuretic, full-bladder analysis of the 82 kidneys studied, we classified 40 kidneys as obstructed, 16 as undetermined, and 26 as unobstructed. In the post-diuretic, empty-bladder analysis, 40 of the 82 kidneys were classified as obstructed. Among those 40 kidneys, the classification changed in 14, of which 11 came to be classified as undetermined and 3 came to be classified as unobstructed (Figures 1 and 2). In addition, of the 16 kidneys that were classified as indeterminate in the post-diuretic, full-bladder images, 13 were classified as unobstructed in the post-diuretic, empty-bladder analysis.

As can be seen in Table 1, the proportional elimination of the radiopharmaceutical was comparable between the full-bladder and empty-bladder analyses, among the female subjects (47.8% ± 24.6% vs. 57.4% ± 20.7%) and among the male subjects (40.4% ± 31.2% vs. 51.6% ± 29.9%), with no significant difference between the two analyses (*p* = 0.8237).

**Table 1:** Descriptive analysis of the proportional elimination of the radiopharmaceutical with a full bladder and with an empty bladder, stratified by gender.

Gender	Number of patients	Bladder status	Mean (%)	Standard deviation (%)	Minimum (%)	Median (%)	Maximum (%)
Female	43	Full	47.84	24.60	-15.05	56.72	83.60
		Empty	57.42	20.76	-2.13	62.55	88.36
Male	39	Full	40.44	31.24	-57.14	46.04	78.30
		Empty	51.65	29.97	-52.94	57.30	87.36

There was no significant difference between the fullbladder and empty-bladder analyses in terms of the proportional elimination of the radiopharmaceutical when adjusted for patient age as a continuous variable (*p* = 0.4733). Table 2 shows the proportional elimination of the radiopharmaceutical when patient age was evaluated as a dichotomous variable. The proportional elimination of the radiopharmaceutical was higher among the subjects < 5 years of age than among those ≥ 5 years of age, in the full-bladder and empty-bladder analyses (28.0% ± 41.6% and 44.7% ± 38.3%, respectively, vs. 47.4% ± 23.9% and 56.5% ± 22.3%, respectively), although the difference between the two groups was not statistically significant (*p* = 1.888).

**Table 2:** Descriptive analysis of the proportional elimination of the radiopharmaceutical with a full bladder and with an empty bladder, stratified by patient age.

Age	Number of patients	Bladder status	Mean (%)	Standard deviation (%)	Minimum (%)	Median (%)	Maximum (%)
≤ 5 years	13	Full	28.08	41.63	-57.14	44.95	69.62
		Empty	44.71	38.30	-52.94	57.06	82.65
> 5 years	69	Full	47.38	23.87	-16.53	50.28	83.60
		Empty	56.55	22.29	-10.15	61.90	88.36

## DISCUSSION

Dynamic scintigraphy with ^99m^Tc-DTPA is currently considered the noninvasive method of choice for the detection of UTO ^(18)^. UTO can cause recurrent infection, diminished renal function, a progressive loss of nephrons, and atrophy of the renal parenchyma^(2)^. DRS makes it possible to estimate two aspects of renal function: clearance and excretion. Clearance is assessed according to extraction of the radiotracer from the blood, whereas excretion is assessed according to the elimination of the radiotracer from the kidneys. The evaluation of diuresis in DRS should be made carefully, considering factors that could influence the outcome^(2,19)^, such as the degree of obstruction, dilation of the renal pelvis, and impairment of renal function; the volumetric capacity of the pelvis, ureter, and bladder; the level of hydration; the positioning and movement of the patient; the timing of administration of the diuretic; the method of interpretation; and the fullness of the bladder.

Since the initial studies of DRS, the influence of bladder fullness has been noted^(18,19)^. A full bladder exerts back pressure on the upper urinary tract, and excretion of the radiopharmaceutical by the kidneys can therefore be significantly reduced^(18)^. In those cases, simply emptying the bladder allows the material to exit the renal pelvis and ureter, making the radiopharmaceutical excretion data more reliable^(18)^.

Various studies using T½ quantification have determined that routine bladder catheterization should be used in all cases^(15)^. In such studies, the catheterization was performed after infusion of the drug, in order to avoid the influence of bladder fullness, as well as to reduce the amount of radiation absorbed by the bladder and gonads^(8)^. However, it is an invasive procedure that causes patient discomfort and predisposes to urinary infection. The standard post-micturition image acquisition protocol does not call for bladder catheterization, which is therefore rarely performed in most nuclear medicine departments. The use of bladder catheterization is advisable only in certain cases, such as those of neurogenic bladder. In addition, bladder catheterization can be postponed until after administration of the diuretic and the subsequent urination, even then being reserved for only those cases in which there is no spontaneously emptying of the bladder^(19)^. It is also important to require the patient to stand after each static urination before and after administration of the diuretic, because additional elimination of the radiopharmaceutical can occur due to the effect of gravity^(19)^.

Post-micturition or post-bladder catheterization images should always be acquired. A diuretic should be administered only when there is persistent retention of the radiopharmaceutical in the excretory tract should a diuretic be administered, after which new dynamic and static images should be acquired before and after bladder emptying.

Various techniques for quantifying the renal transport of the radiopharmaceutical have been proposed^(19)^. One such method considers the excretory pathways to be obstructed when the T½ obtained from the post-diuretic excretion curve is > 20 min. That method is valid only if the entire study is performed with a catheterized bladder, in order to eliminate the influence of bladder fullness. When bladder catheterization is not routinely used, only the post-micturition images can be quantified without interference from the influence of bladder fullness. In such cases, an analysis derived from the T½ method-in which the kidney is considered obstructed when the post-diuretic excretion is < 50% in 20 min, corresponding to a T½ > 20 min-can be used. Similarly, an unobstructed kidney shows a post-diuretic excretion of ≥ 60% in 20 minutes (T½ < 15 min) and the result is considered indeterminate when the post-diuretic excretion is 50-60%^(12)^.

Although many nuclear medicine departments use dynamic renal study protocols that do not call for the acquisition of post-micturition images, our data demonstrate the importance of considering the influence of bladder fullness in the interpretation of the results. The importance of that influence was underscored-numerically being even greater, although the difference was not statistically significant-in children ≤ 5 years of age, among whom which the diagnosis of UTO is often more difficult^(8)^. The acquisition of post-micturition images, even if it increases the length of the patient visit, can increase the accuracy of the interpretation of the examination results, reducing the number of false-positive results for obstruction.

The main limitations of this study include the biases inherent to a retrospective study and the fact that patient follow-up was not performed in order to determine the sensitivity and specificity of the method used. However, given that dynamic scintigraphy with a diuretic was originally described and independently validated with an empty bladder^(8,20)^, the present study aimed to discuss only the influence of bladder fullness in the application of the method.

## CONCLUSION

During a DRS examination with diuretic administration, it is fundamental to acquire post-micturition images for the accurate and reliable analysis of the proportional elimination of the radiopharmaceutical from the kidney. Thus, false-positive results for UTO can be avoided.
